# A rare mixed ovarian tumor associating borderline endometrioid component and borderline serous component

**DOI:** 10.11604/pamj.2022.41.73.30418

**Published:** 2022-01-26

**Authors:** Seifeddine Ben Hammouda, Manel Njima

**Affiliations:** 1 Department of Pathology, Fattouma Bourguiba University Hospital, Monastir, Tunisia,; 2 Faculty of Medicine, University of Monastir, Monastir, Tunisia

**Keywords:** Ovary, borderline tumor, endometriosis

## Image in medicine

A 37-year-old woman was admitted to the department of Gynecology and Obstetrics for management of ovarian cyst, for which a left oophorectomy was performed. The specimen was evaluated then in the department of pathology. On gross examination, the ovarian cyst had a smooth surface and measured 5.5x4.5x 2.5cm. Cut section revealed that the cyst was multilocular, filled with clear fluid and containing several buds. Microscopically, the cyst consisted in a biphasic tumor associating borderline endometrioid component, composed of confluent glands of endometrioid-type epithelium with mild atypical nuclei and borderline serous component. This serous component was made by numerous bulbous, irregularly contoured papillae with fibrous cores and lined by pseudostratified columnar cells with moderate atypia. Immunohistochemical study using WT1 showed positive labeling in the borderline serous component (blue asterisk) and absence of labeling in the borderline endometrioid component (red asterisk). The diagnosis of a mixed ovarian tumor associating a borderline endometrioid component and a borderline serous component was given. A mixed tumor associating two types of ovarian borderline tumors is exceptionally described in the literature. In our case, the presence of a borderline endometrioid component associated with a borderline serous component raises the role of endometriosis in the genesis of ovarian tumors. The pathogenetic transformation from endometriosis to ovarian tumors is not fully understood, but it seems mainly related to many factors: inflammation, oxidative stress, hyperestrogenism, and specific genomic alterations.

**Figure 1:**
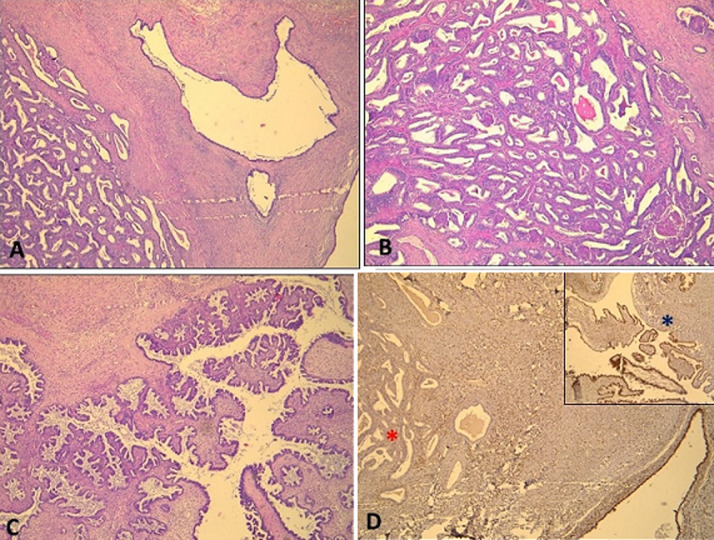
A) histological findings showing biphasic tumor (HEx100); B) associating borderline endometrioid component (HEx200); C) and borderline serous component (HEx200); D) immunoreactivity with WT1 in the borderline serous component (blue asterisk) and absence of expression in the endometrioid component (red asterisk)

